# Liquid Crystalline Esters of Dibenzophenazines

**DOI:** 10.3390/ma8010270

**Published:** 2015-01-14

**Authors:** Kevin John Anthony Bozek, Kuan I Ho, Tom Saint-Martin, Panos Argyropoulos, Vance E. Williams

**Affiliations:** 1 Department of Chemistry, Simon Fraser University, Burnaby, BC V5A 1S6, Canada; E-Mails: kbozek@sfu.ca (K.J.A.B.); kiho@sfu.ca (K.IH.); tom.saint-martin@outlook.fr (T.S.M.); pargy091@uottawa.ca (P.A.); 2 Department of Chemistry, Université Pierre & Marie Curie, Paris 75252, France

**Keywords:** dibenzophenazine, discotic, columnar liquid crystal

## Abstract

A series of esters of 2,3,6,7-tetrakis(hexyloxy)dibenzo[*a*,*c*]phenazine-11-carboxylic acid was prepared in order to probe the effects of the ester groups on the liquid crystalline behavior. These compounds exhibit columnar hexagonal phases over broad temperature ranges. Variations in chain length, branching, terminal groups, and the presence of cyclic groups were found to modify transition temperatures without substantially destabilizing the mesophase range.

## 1. Introduction

Discotic liquid crystals (DLCs) have been the focus of intense scrutiny over the past several years, owing in large part to their usefulness as organic semiconductors with high charge carrier mobilities, a lack of grain boundaries, and a predisposition to uniformly align. Such properties make DLCs attractive candidate materials for light emitting diodes, photovoltaic devices, and field effect transistors [1,2,3,4,5,6]. Successful exploitation of discotic liquid crystals requires balancing molecular electronic properties with the requirements of self-assembly. In principle, this can be accomplished by creating series of molecules in which phase behavior can be fine-tuned without substantially altering the electronic properties of the building blocks. Ideally, such families would also be constructed from readily available starting materials via reliable and facile synthetic approaches.

With these criteria in mind, we undertook the synthesis of a series of dibenzophenazine esters **2**–**6**. Because esters can be formed from the reaction of an appropriate acid (e.g., **1**) with any of number of readily available alcohols, discotic esters, as a family, hold the promise for considerable structural diversity. In the present work, we explore the impact of varying the R group with linear (**2**), branched (**3**) cyclic (**4**–**5**) and hydroxy-terminated substituents (**6**). Because communication across the ester group is limited, these structural changes are expected to have only a minimal impact on the electronic properties of the discotic core while allowing for the fine-tuning of the LC phase range. Surprisingly, despite these potential advantages, discotic esters have been largely neglected in the literature [7,8,9,10]. Dibenzophenazine esters in particular were targeted because of our previous observation that the methyl ester **2a** possesses a columnar hexagonal phase over an extremely broad temperature range (111–201 °C) [11]. Because of the breadth of this phase, we posited that replacing the methyl group would lead to changes in phase stability without completely destroying the liquid crystallinity.

## 2. Results and Discussion

**Scheme 1:**
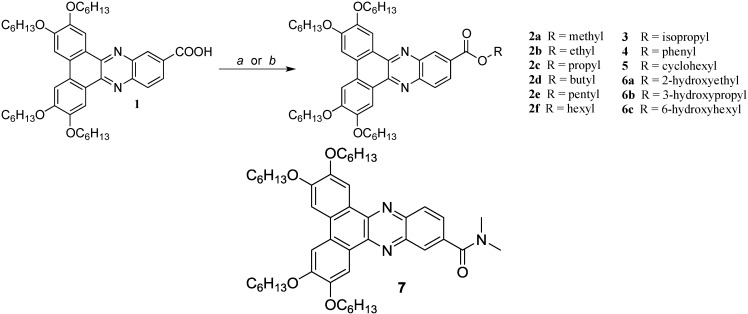
Reagents and conditions: (a) oxalyl chloride, dimethylformamide, dichloromethane, room temperature (RT), 3 h; ROH, pyridine, dichloromethane, RT, 20 h; (b) 4-dimethylaminopyridine (DMAP), 1-ethyl-3-(3-dimethylaminopropyl)carbodiimide (EDCI), dichloromethane, corresponding alcohol, RT, 24 h.

The synthesis of esters **2b**–**f** and **3**–**6** is outlined in Scheme 1. The linear alkyl esters **2b**–**f** and hydroxy-terminated esters **6a** and **6c** were prepared according to the method previously reported for **6b** [12]. The acid **1** was first converted to its acid chloride using oxalyl chloride in the presence of dimethylformamide; subsequent treatment of this acid chloride with an excess of the appropriate alcohol afforded the target esters in moderate to good yields (33%–85%) after final purification.

Attempts to prepare the branched isopropyl analog **3** via this route were unsuccessful; rather than isolating this ester, the dimethylamide **7** was formed, presumably from the reaction of residual dimethylamine present in the DMF. Although not specifically targeted, this amide was isolated in sufficient yields to allow us to examine its phase behavior. In order to access the branched ester **3**, we adopted an alternative synthetic approach in which the acid was activated with 1-ethyl-3-(3-dimethylaminopropyl)carbodiimide (EDCI)/4-dimethylaminopyridine (DMAP), followed by reaction with isopropanol. This improved synthetic route was also used to prepare the cyclic esters, **4** and **5**, which were isolated in 70% and 33% yields, respectively.

**Figure 1:**
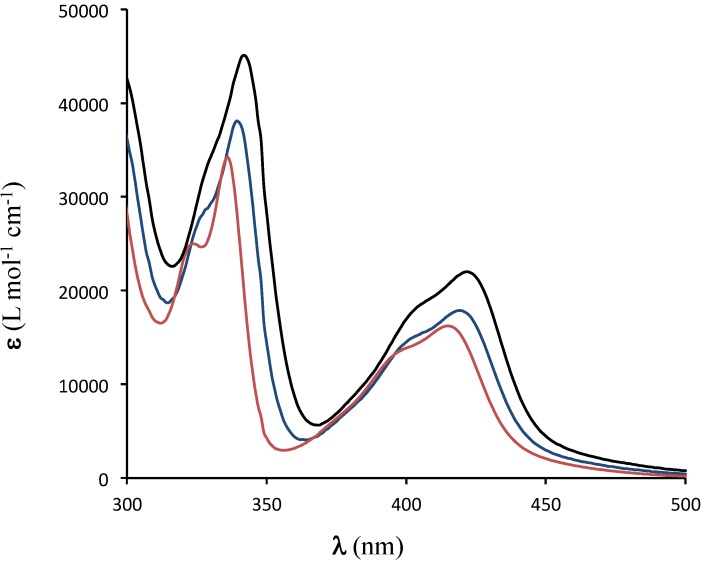
UV-visible absorption spectra of **2b** (blue), **4** (black) and **7** (red) in CHCl_3_.

The electronic properties of all compounds were examined by UV-visible absorption spectroscopy in dilute chloroform solutions. The spectra of all esters are similar, with the λ_max_ of the longest wavelength absorption band falling in the range of 419–422 nm; this small variation is unsurprising, since the electronic structure of the core is not expected to be sensitive to the nature of the functional group on the ester. A second, unresolved peak is also observed at slightly shorter wavelengths (λ_max_ ~ 405 nm). Both peaks are slightly blue-shifted to λ_max_ = 414 nm and 395 nm in the spectrum of the amide **7** (Figure 1), presumably because of differing electron-withdrawing ability of this substituent [13].

**Figure 2:**
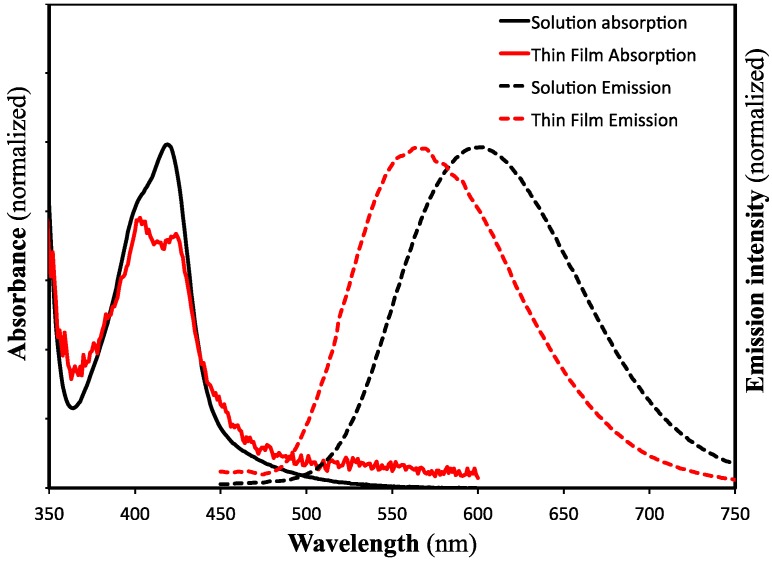
UV-visible absorption and emission spectra of **2a** in solution (10^−5^ M CHCl_3_) and as thin films. Emission spectra were obtained using an excitation wavelength of 420 nm.

The optical properties of the esters were further probed by examining the absorption and emission spectra of the methyl ester, **2a**, in solution and when cast as a thin film (Figure 2). The ester **2a** exhibits strong luminescence in both the solution and the solid state, although we have not measured quantum yields at this time. The solid state and solution absorption spectra of this compound are very similar to one another, although the two long wavelength absorption bands noted above are sharper and more resolved in the thin film. Much larger changes are observed between the emission spectra. In solution, **2a** exhibits a single broad peak centered at 600 nm; this peak is blue-shifted by approximately 40 nm in the solid state. The excitation spectra (not shown) of both samples closely match the absorption profiles. The solution emission and absorption spectra of **2a** closely resemble those previously reported by Szydłowska and coworkers for the carboxylic acid **1** [14].

**Figure 3:**
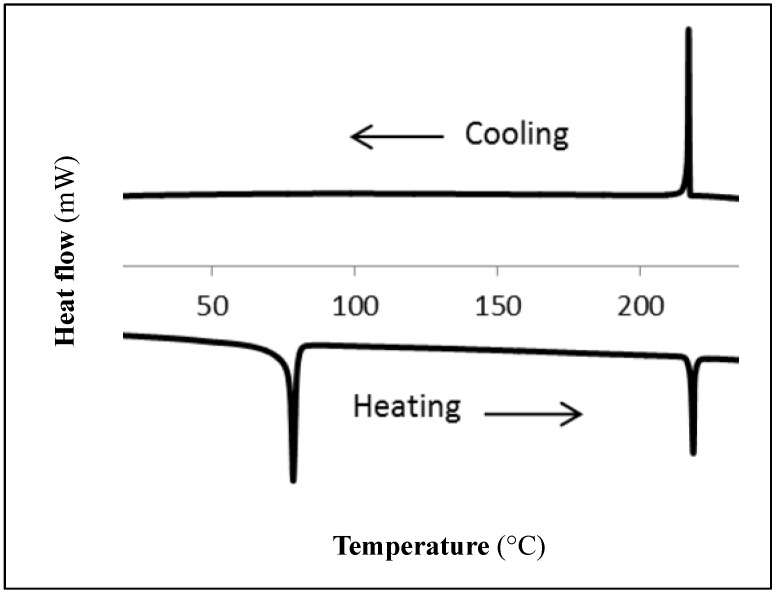
Differential scanning calorimetry (DSC) endotherm and exotherm for first heating and cooling cycle of **3** (heating/cooling rate = 10 °C/min).

The liquid crystal properties of compounds **2**–**7** were studied by polarized optical microscopy (POM), differential scanning calorimetry (DSC), and variable temperature X-ray diffraction (XRD) experiments, the results of which are summarized in Table 1 and Table 2. DSC endotherms of all compounds exhibited at least two phase transitions upon first heating: a large enthalpy transition at low temperature followed by a smaller enthalpy transition at higher temperatures, consistent with crystalline-to-liquid crystalline melting and liquid crystalline (LC)-to-isotropic clearing transitions, respectively. The DSC cooling curves for all compounds show the isotropic-to-LC transition with minimal hysteresis. Notably, the LC-solid transitions are absent in all cases, indicating that the mesophases supercool from their LC phases (Figure 3). Second heating curves obtained immediately following cooling runs generally either lack a melting peak, or exhibit an exothermic peak associated with cold-crystallization of the sample. In contrast, cooled DSC samples that have been allowed to sit for extended periods do exhibit melting peaks on heating, providing evidence that samples crystallize over time.

**Figure 4:**
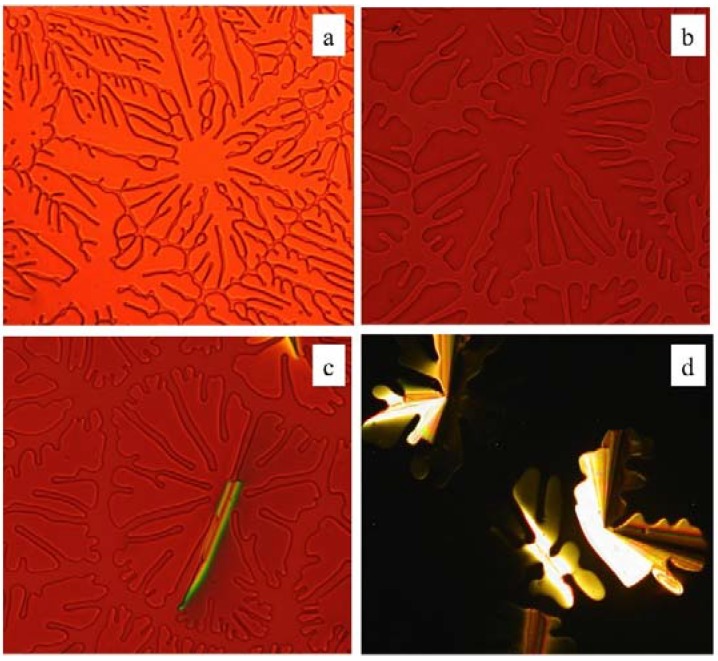
Representative polarized optical micrographs of columnar phases formed upon cooling from the isotropic phase of (**a**) **2b**; (**b**) **4**; (**c**) **6c** and (**d**) **7** (200× magnification). Images (**a**)–(**c**) viewed employing a 530 nm quarter wavelength retardation plate in order to improve viewing contrast.

**Figure 5:**
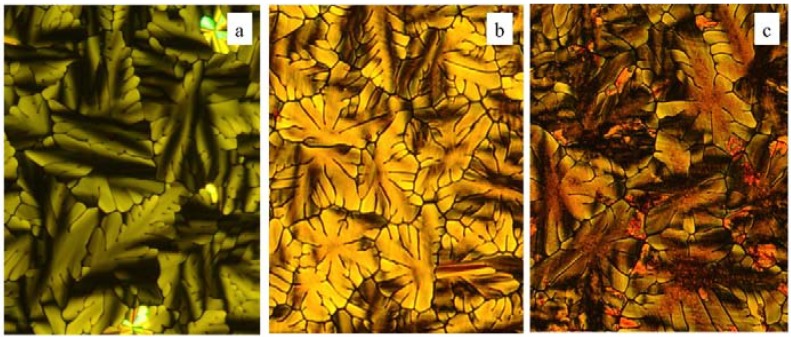
Polarized optical microscopy (POM) micrographs of compound **4** (**a**) at 175 °C; (**b**) at room temperature after 7 days and (**c**) at room temperature after 27 days.

Examination of **2**–**7** by microscopy confirms that all compounds form liquid crystalline phases on both heating and cooling. Samples cooled slowly into their LC phase from the isotropic state exhibit dendritic textures (Figure 4) that are typical of columnar hexagonal (Col_h_) phases. These samples show a strong tendency to homeotropically align between untreated glass substrates when slowly cooled from their isotropic liquids, which is a common feature of dibenzophenazine discotic mesogens [15,16]. The liquid crystalline textures of all samples are retained down to room temperature, well below their melting points, consistent with the DSC observation of supercooling. Most of the compounds were observed to crystallize at room temperature over the course of approximately 30 min. A notable exception was the phenyl ester **4**, which crystallizes over a period of weeks rather than minutes (Figure 5). It is not clear at this time why this compound crystallizes at a much slower rate than the other esters studied, but does suggest that variations in the ester group may provide a practical means to kinetically suppress crystallization.

**Table 1:** Phase behavior of compounds 2–7. ^a^ Transition temperatures and enthalpies were determined by differential scanning calorimetry (DSC) (scan rate = 10 °C/min) on second heating run (see text); ^b^ Cr = crystal, Col_h_ = columnar hexagonal, I = isotropic; ^c^ see reference 11; ^d^ see reference 12.

Compound	Phase	*T*/°C (Δ*H*/J g^−1^) ^a^	Phase	*T*/°C (Δ*H*/J g^−1^) ^a^	Phase ^b^
**2a** ^c^	Cr	111.3 (39.1) 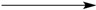	Col_h_	200.6 (4.7) 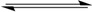 196.5 (4.8)	I
**2b**	Cr	64.3 (45.6) 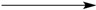	Col_h_	210.8 (8.0) 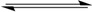 209.5 (11.1)	I
**2c**	Cr	86.8 (40.1) 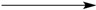	Col_h_	212.1 (7.9) 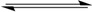 210.2 (9.8)	I
**2d**	Cr	92.5 (42.0) 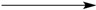	Col_h_	209.6 (8.3) 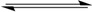 208.2 (8.6)	I
**2e**	Cr	97.5 (68.0) 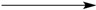	Col_h_	202.9 (7.7) 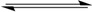 202.0 (7.7)	I
**2f**	Cr	92.7 (61.8) 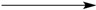	Col_h_	191.3 (4.6) 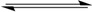 190.9 (6.3)	I
**3**	Cr	78.6 (32.7) 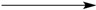	Col_h_	218.8 (9.6) 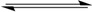 217.1 (10.6)	I
**4**	Cr	124.7 (38.7) 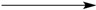	Col_h_	220.5 (4.1) 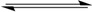 218.6 (5.8)	I
**5**	Cr	84.7 (18.5) 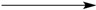	Col_h_	202.5 (5.6) 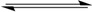 199.1 (7.3)	I
**6a**	Cr	99.7 (15.0) 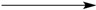	Col_h_	198.7 (6.6) 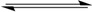 197.6 (6.5)	I
**6b** ^d^	Cr	130.1 (71.8) 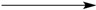	Col_h_	202.0 (9.4) 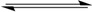 201.1 (9.3)	I
**6c**	Cr	96.5 (51.2) 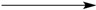	Col_h_	188.8 (7.9) 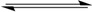 187.8 (8.8)	I
**7**	Cr	122.9 (45.5) 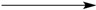	Col_h_	180.3 (2.9) 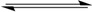 180.1 (3.1)	I

**Table 2:** X-ray diffraction (XRD) data and lattice constants for compounds **2**–**7**.

Compound	Temperature (°C)	d-spacings (Å)	Miller Index (hkl)	Phase (Lattice Constants)
**2a**	150	17.3	100	Col_h_
		4.3	Alkyl Halo	(*a* = 20.0 Å)
		3.5	π–π stacking	–
**2b**	179	17.7	100	Col_h_
		5	Alkyl Halo	(*a* = 20.5 Å)
		3.8	π–π stacking	–
**2c**	177	17.4	100	Col_h_
		4.9	Alkyl Halo	(*a* = 20.1 Å)
**2d**	178	17.7	100	Col_h_
		4.9	Alkyl Halo	(*a* = 20.5 Å)
**2e**	179	17.9	100	Col_h_
		4.7	Alkyl Halo	(*a* = 20.7 Å)
		3.6	π–π stacking	–
**2f**	155	18.5	100	Col_h_
		4.6	Alkyl Halo	(*a* = 21.4 Å)
**3**	185	17.7	100	Col_h_
		10.3	110	(*a* = 20.4 Å)
		5.4	Alkyl Halo	–
**4**	175	17.7	100	Col_h_
		4.5	Alkyl Halo	(*a* = 20.4 Å)
**5**	155	18.1	100	Col_h_
		10.6	110	(*a* = 20.9 Å)
		6.6	Alkyl Halo	–
**6a**	160	17.3	100	Col_h_
		5.1	Alkyl Halo	(*a* = 20.0 Å)
		3.6	π–π stacking	–
**6b**	170	17.5	100	Col_h_
		4.6	Alkyl Halo	(*a* = 20.2 Å)
		3.5	π–π stacking	–
**6c**	157	18.3	100	Col_h_
		5.1	Alkyl Halo	(*a* = 21.1 Å)
**7**	169	17.1	100	Col_h_
		4.7	Alkyl Halo	(*a* = 19.8 Å)

XRD experiments are consistent with the assignment of Col_h_ phases being formed for all mesogens. X-ray diffraction patterns of the liquid crystalline phases of all compounds exhibit one intense peak in the low angle region, which was identified as the (100) peak of a hexagonal lattice. A second broad alkyl halo peak at distances of approximately 4.5 Å is also observed in all cases. Peaks associated with the π–π stacking distances at approximately 3.5 Å were also observed in the cases of compounds **2a**, **2b**, **2e**, **6a** and **6b**. Changes in the ester group have only a small impact on the intercolumnar spacing (Table 2).

**Figure 6:**
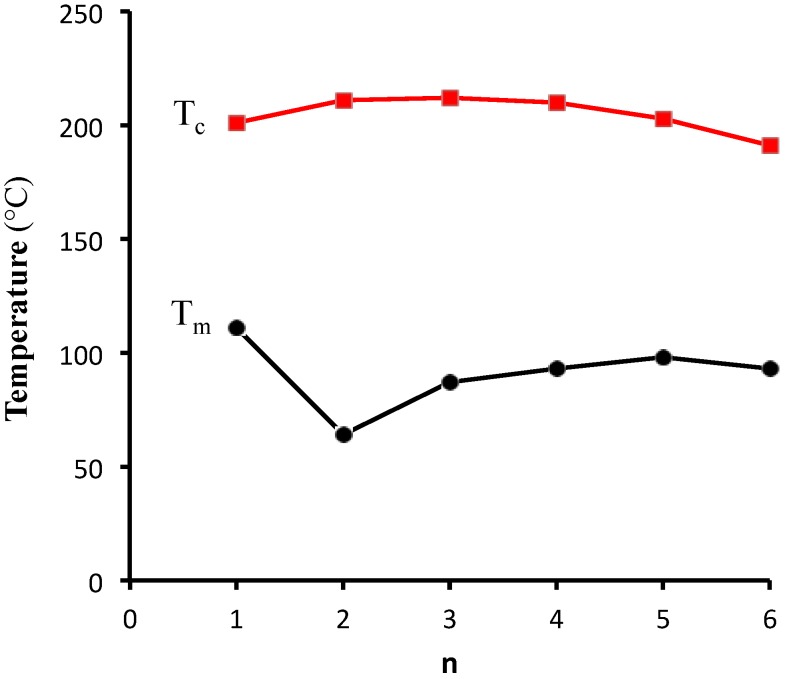
Plot of melting (*T*_m_, black circles) and clearing (*T*_c_, red squares) temperatures of linear alkyl esters **2a**–**f**, as a function of ester chain length, where *n* = #carbons in alkyl chain of ester (*i.e.*, **2a**, *n* = 1; **2b**, *n* = 2,* etc.*).

The effects of chain length on the LC phase ranges of the linear esters **2a**–**f** are shown graphically in Figure 6. Increasing chain length is accompanied by a general tendency towards lower clearing temperatures and higher melting temperatures, resulting in an overall narrowing of the LC range, from a maximum 137 °C range for **2b** (64–211 °C) to a 98 °C range for **2f** (93–191 °C). This effect parallels the typical trend for discotic mesogens when one or more of the peripheral side chains are varied [17,18]. In the present case, the narrowing of the phase range is much less pronounced than when all of the side chains of a discotic are lengthened. The methyl ester **2a** represents an anomaly within this series, with a melting temperature that is almost 50 °C higher than that of the ethyl ester **2b**; as a consequence, this molecule has the narrowest LC phase range in this family. Thus, extending the ester chain length beyond a single carbon has the beneficial effect of shifting the LC phase range closer to room temperature.

The introduction of branched chains into discotic mesogens is often associated with significant changes in melting and clearing temperatures. However, the nature of these changes is far from consistent; both elevated and depressed transition temperatures are found to accompany branching [19,20,21]. It was therefore of interest to compare the differences between the isopropyl derivative **3** with the linear esters in series **2**. Of particular note, the clearing temperature of the branched ester **3** (219 °C) is appreciably higher than that of its linear isomer **2c** (*T*_c_ = 212 °C), indicating that branching leads to a stabilization of the LC phase. In contrast, the melting temperature of the linear ester **2c** (87 °C) is higher than that of **3** (79 °C). This may reflect either the greater stability of the LC phase of **3** (which would lead to a depression of the melting point) or, as seems more likely, the destabilization of its crystalline solid phase. More generally, the branched-chain derivative **3** exhibits one of the lowest melting temperatures and highest clearing points of any of the esters studied in this work, with only the ethyl compound **2b** having a broader LC phase range. This stabilization of the LC phase may result from the isopropyl group being able to fill space more effectively, allowing for tighter packing and stronger interactions [22].

Replacing the linear and branched alkyl groups in **2** and **3** with a cyclohexyl group in **5** led to only modest changes in phase behavior. Despite the bulkiness of this group, **5** both melts and clears at temperatures that are similar to the members of series **2** and **3**, and it appears that the cyclohexyl group neither strongly stabilizes nor destabilizes the LC phase. In marked contrast, both transition temperatures of the phenyl ester **4** are substantially elevated, with melting and clearing temperatures that are 40 °C and 18 °C higher, respectively, than those of the structurally similar derivative **5**. Indeed, **4** has the highest *T*_m_ and *T*_c_ values of any of the compounds prepared for this study, denoting that both the crystalline and liquid crystalline phases are stabilized by the phenyl substituent, likely via π–π interactions.

**Figure 7:**
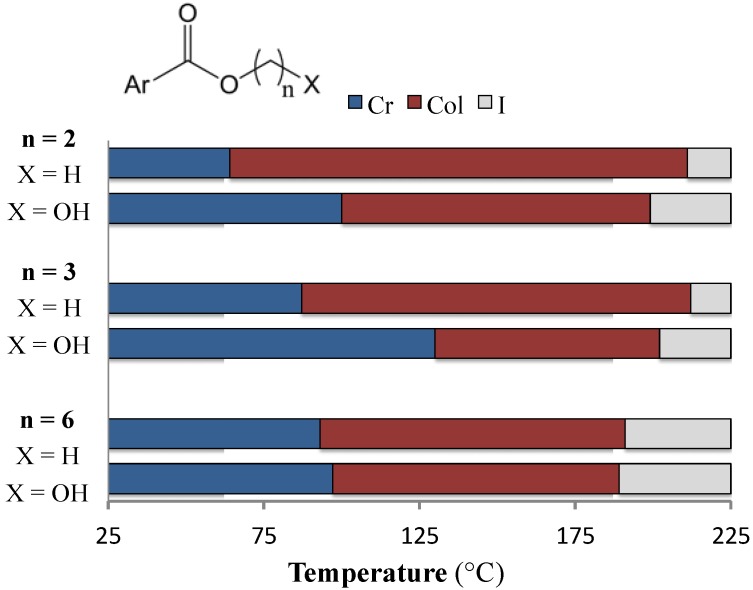
Phase ranges of compounds **2b**, **2c**, **2f** and **6a**–**c**; Cr = crystalline solid, Col = columnar hexagonal LC, I = isotropic liquid.

We had anticipated that incorporating chains terminated with hydroxyl groups (**6a**–**c**) would lead to a stabilization of the liquid crystalline phases, as is often the case for mesogens capable of hydrogen bonding [11,23,24]. Surprisingly, replacing the terminal hydrogen of an alkyl chain with a hydroxyl group, rather than increasing the LC-isotropic transition temperature, actually leads to a slight decrease in *T*_c_ (Figure 7). Thus, it appears that hydrogen bonding does little to stabilize the LC phases in the present systems, and may in fact destabilize these phases. Similar trends have also been observed for triphenylene and penta-ethynylbenzene discotic mesogens bearing a single hydroxy-terminated chain [25,26].

Finally, the amide derivative **7** also forms a columnar hexagonal phase, albeit with a clearing temperature (180 °C) that is substantially lower than any of the esters studied. We ascribe this depression of the isotropization temperature to differences in the electronic properties between esters and amides, with amides being less electron-withdrawing (Hammett σ_p_ = 0.37) than esters (σ_p_ = 0.45) [13]. This observation is consistent with our earlier finding of a strong correlation between clearing temperatures of discotic mesogens and the electron-withdrawing ability substituents directly attached to the dibenzophenazine core [27]. It is notable that changing the functional group from an ester to an amide has a much greater tendency to destabilize the columnar phase than changing the nature of the ester. Thus, electronic effects appear to trump steric considerations, at least over the range of functionalities examined.

## 3. Experimental Section

All solvents employed were reagent grade and were used as obtained from suppliers unless otherwise noted. Oxalyl chloride was purchased from Aldrich (St. Louis, MO, USA). The 1,3-propanediol was purchased from ICN biomedical supplies (Santa Ana, CA, USA). Pyridine was purchased from Anachemia (Richmond, Canada) and was dried over KOH pellets. The DMAP and EDCI were purchased from TCI (Portland, OR, USA). Column chromatography was performed using silica gel 60 (230–400 mesh) purchased from Silicyle Inc. (Quebec City, Canada). The carboxylic acid derivative, **1** [11] and esters **2a** [11] and **6b** [12] were prepared according to previously published methods.

The 400 MHz ^1^H NMR spectra were obtained using a Bruker AMX-400 400MHz NMR spectrometer (Billerica, MA, USA). 500 MHz ^1^H NMR spectra and 125 MHz ^13^C NMR spectra were obtained using a Varian AS500 Unity Inova 500 MHz spectrometer (Billerica, MA, USA). The 600 MHz ^1^H NMR and 150 MHz ^13^C NMR spectra were obtained using a Bruker Avance 600 MHz spectrometer equipped with a TCI cryoprobe. Phase transition temperatures and enthalpies were investigated using differential scanning calorimetry on a TA Instruments DSC Q2000 (New Castle, DE, USA) equipped with a TA Instruments Refrigerated Cooling System 90, heating and cooling at a rate of 10 °C min^−1^. Texture analysis was carried out using optical polarizing microscopy on an Olympus BX50 microscope (Richmond Hill, Canada) with crossed polarizers using a Linkam LTS350 heating stage (Tadworth, UK). X-ray scattering experiments were conducted using a Rigaku R-Axis Rapid diffractometer (The Woodlands, TX, USA) equipped with a temperature controller [28]. X-ray samples were prepared by first heating the compound to its isotropic phase, then loading it by capillary action into a glass capillary.

Infrared spectroscopy (IR) spectra were obtained using a Perkin-Elmer Spectrum One FT-IR with an attenuated total reflectance (ATR) attachment. High resolution mass spectroscopy was acquired on a BrukermicrOTOF-Q II using electrospray ionization positive at the University of Notre Dame. UV-Vis spectra were obtained using a Varian Cary 300 Bio UV-Visible spectrometer (Santa Clara, CA, USA) and fluorescence measurements were obtained using a PTI QuantaMaster Fluorescence Spectrometer (London, ON, Canada). Thin films used for UV-visible and emissions studies of **2a** were prepared by spin coating (1700 rpm) a 10^−3^ M chloroform solution onto a microscope cover slip using a Laurell WS-400A-6NPP/LITE spin coater (North Wales, PA, USA).

### 3.1. Synthesis Method 1 (Oxalyl Chloride Method)

In a round bottom flask (100 mL) equipped with a magnetic stir bar, the carboxylic acid (1 equivalent) was suspended in dry DCM (50 mL) and the mixture was set to stir. To this was added DMF (0.1 equivalent) and oxalyl chloride (0.1 mL 2 M solution, 1.1 equivalent). The resulting suspension was allowed to stir for 2.5 h at room temperature. At this time the solvent was removed under vacuum. The solid was then resuspended in dry DCM (25 mL) and a mixture of alcohol (10 mL, 30 equivalent) and pyridine (10 mL, 30 equivalent) in dry DCM (40 mL) was added and the reaction was allowed to stir overnight at room temperature. The resulting suspension was filtered and washed with water (2 × 50 mL), HCl (10%, 50 mL), and brine (50 mL). The organic layers were combined, dried over MgSO_4_, and the solvent was removed under vacuum affording the target compound.

### 3.2. Synthesis Method 2 (EDCI/DMAP Method)

In a round bottom flask (100 mL) equipped with a magnetic stir bar, the carboxylic acid (1 equivalent), the alcohol (1.1 equivalent), and DMAP (3 equivalent) were dissolved in dry DCM (25 mL) and the mixture was set to stir and cooled to 0 °C in an ice water bath. To this was added EDCI (2 equivalent). The resulting mixture was allowed to stir for 24 h. The resulting solution was washed with water (2 × 50 mL), HCl (10%, 50 mL), and brine (50 mL). The organic layers were combined, dried over MgSO_4_, and the solvent was removed under vacuum to afford the target compound.

Compound **2b** was prepared according to Method 1 and purified by column chromatography using dichloromethane as eluent (*R*_f_ = 0.43) to afford orange crystals in 36% yield and then further purified via recrystallization from hexanes. ^1^H NMR (400 MHz, CDCl_3_): δ 0.95 (m, 14H), 1.42 (m, 15H), 1.59 (m, 10H), 1.98 (m, 8H), 4.26 (t, 4H, *J* = 6.6 Hz), 4.33 (dd, 4H, *J* = 12.1, 6.5 Hz), 4.52 (q, 2H, *J* = 7.1 Hz), 7.66 (m, 2H), 8.34 (m, 2H), 8.73 (m, 2H), 9.0 (d, 1H, *J* = 1.6 Hz) ppm; ^13^C NMR (151 MHz, CDCl_3_): δ 170.80, 152.08, 152.01, 149.49, 142.65, 142.60, 141.63, 140.66, 136.35, 129.68, 127.78, 127.73, 126.85, 126.72, 123.62, 123.58, 108.88, 108.79, 106.45, 106.43, 69.64, 69.19, 69.15, 39.81, 35.54, 31.67, 29.71, 29.32, 29.25, 25.85, 25.82, 22.66, 14.07, 14.05; UV (λ_max_(log ε), CHCl_3_): 288(4.58), 339(4.47), 419(4.44); IR (neat): 1723 cm^−1^ (ν: C=O); High resolution mass spectrometry (HRMS) (Electrospray ionization (ESI))+ : *m*/*z* calcd for C_47_H_65_N_2_O_6_^+^: 753.4838, found: 753.4837.

Compound **2c** was prepared according to Method 1 and purified by column chromatography using dichloromethane as eluent (*R*_f_ = 0.43) to afford orange crystals in 49% yield and then further purified via recrystallization from hexanes. ^1^H NMR (400 MHz, CDCl_3_): δ 0.95 (m, 16H), 1.42 (m, 15H), 1.59 (m, 10H), 1.98 (m, 8H), 4.29 (t, 4H, *J* = 6.5 Hz), 4.36 (dd, 4H, *J* = 12.3, 6.4 Hz), 4.42 (t, 2H, *J* = 6.6 Hz), 7.74 (s, 2H), 8.37 (m, 2H), 8.81 (d, 2H, *J* = 4.1 Hz), 9.04 (d, 1H, *J* = 1.6 Hz) ppm; ^13^C NMR (151 MHz, CDCl_3_): δ 166.29, 152.32, 152.06, 149.56, 149.46, 143.38, 143.22, 142.77, 140.58, 132.03, 130.41, 129.27, 128.24, 127.19, 126.68, 123.57, 123.46, 108.99, 108.79, 106.45, 106.32, 69.66, 69.59, 69.18, 69.16, 61.45, 31.67, 29.33, 29.31, 29.25, 25.85, 25.82, 22.67, 22.66, 14.45, 14.07, 14.05; UV (λ_max_(log ε), CHCl_3_): 288(4.85), 339(4.75), 419(4.41); IR (neat): 1726 cm^−1^ (ν: C=O); HRMS (ESI+): *m*/*z* calcd for C_48_H_67_N_2_O_6_^+^: 767.4994, found: 767.4994.

Compound **2d** was prepared according to Method 1 and purified by column chromatography using dichloromethane as eluent (*R*_f_ = 0.43) to afford orange crystals in 36% yield and then further purified via recrystallization from hexanes. ^1^H NMR (400 MHz, CDCl_3_): δ 0.95 (m, 18H), 1.42 (m, 15H), 1.59 (m, 10H), 1.98 (m, 8H), 4.29 (t, 4H, *J* = 6.5 Hz), 4.36 (dd, 4H, *J* = 12.3, 6.4 Hz), 4.46 (t, 2H, *J* = 6.6 Hz), 7.74 (s, 2H), 8.35 (m, 2H), 8.81 (d, 2H, *J* = 4.1 Hz), 9.04 (d, 1H, *J* = 1.6 Hz) ppm; ^13^C NMR (151 MHz, CDCl_3_): δ 166.29, 152.32, 152.06, 149.56, 149.46, 143.38, 143.22, 142.77, 140.58, 132.03, 130.41, 129.27, 128.24, 127.19, 126.68, 123.57, 123.46, 108.99, 108.79, 106.45, 106.32, 69.66, 69.59, 69.18, 69.16, 61.45, 31.67, 29.33, 29.31, 29.25, 25.85, 25.82, 22.67, 22.66, 14.45, 14.07, 14.05; UV-vis (λ_max_(log ε), CHCl_3_): 288(4.83), 339(4.72), 419(4.31); IR (neat): 1726 cm^−1^ (ν: C=O); HRMS (ESI+): *m*/*z* calcd for C_49_H_69_N_2_O_6_^+^: 781.515, found: 781.5147.

Compound **2e** was prepared according to Method 1 and purified by column chromatography using dichloromethane as eluent (*R*_f_ = 0.43) to afford orange crystals in 37% yield and then further purified via recrystallization from hexanes. ^1^H NMR (400 MHz, CDCl_3_): δ 0.95 (m, 20H), 1.42 (m, 15H), 1.59 (m, 10H), 1.98 (m, 8H), 4.29 (t, 4H, *J* = 6.5 Hz), 4.36 (dd, 4H, *J* = 11.9, 6.6 Hz), 4.45 (t, 2H, *J* = 6.7 Hz), 7.73 (s, 2H), 8.37 (m, 2H), 8.81 (d, 2H, *J* = 4.6 Hz), 9.04 (d, 1H, *J* = 1.3 Hz) ppm; ^13^C NMR (151 MHz, CDCl_3_): δ 166.29, 152.32, 152.06, 149.56, 149.46, 143.38, 143.22, 142.77, 140.58, 132.03, 130.41, 129.27, 128.24, 127.19, 126.68, 123.57, 123.46, 108.99, 108.79, 106.45, 106.32, 69.66, 69.59, 69.18, 69.16, 61.45, 31.67, 29.33, 29.31, 29.25, 25.85, 25.82, 22.67, 22.66, 14.45, 14.07, 14.05; UV-vis (λ_max_(log ε), CHCl_3_): 288(4.72), 339(4.61), 419(4.28); FT-IR (neat): 1722 cm^−1^ (ν: C=O); HRMS (ESI+): *m*/*z* calcd for C_50_H_71_N_2_O_6_^+^: 795.5307, found: 795.5307.

Compound **2f** was prepared according to Method 1 and purified by column chromatography using dichloromethane as eluent (*R*_f_ = 0.43) to afford orange crystals in 33% yield and then further purified via recrystallization from hexanes. ^1^H NMR (400 MHz, CDCl_3_): δ 0.95 (m, 22H), 1.42 (m, 15H), 1.59 (m, 10H), 1.98 (m, 8H), 4.27 (t, 4H, *J* = 6.6 Hz), 4.34 (dd, 4H, *J* = 11.8, 6.5 Hz), 4.45 (t, 2H, *J* = 6.7 Hz), 7.68 (s, 2H), 8.35 (m, 2H), 8.75 (d, 2H, *J* = 5.3 Hz), 9.01 (d, 1H, *J* = 1.6 Hz) ppm; ^13^C NMR (151 MHz, CDCl_3_): δ 166.29, 152.32, 152.06, 149.56, 149.46, 143.38, 143.22, 142.77, 140.58, 132.03, 130.41, 129.27, 128.24, 127.19, 126.68, 123.57, 123.46, 108.99, 108.79, 106.45, 106.32, 69.66, 69.59, 69.18, 69.16, 61.45, 31.67, 29.33, 29.31, 29.25, 25.85, 25.82, 22.67, 22.66, 14.45, 14.07, 14.05; UV-vis (λ_max_(log ε), CHCl_3_): 288(4.92), 339(4.81), 419(4.48); FT-IR (neat): 1722 cm^−1^ (ν: C=O); HRMS (ESI+): *m*/*z* calcd for C_51_H_73_N_2_O_6_^+^: 809.5463, found: 809.5465.

Compound **7** was prepared according to Method 1 and purified by column chromatography using 1% methanol in dichloromethane as eluent (*R*_f_ = 0.08) to afford orange crystals in 40% yield. ^1^H NMR (400 MHz, CDCl_3_): δ 0.94 (m, 11H), 1.25 (s, 3H), 1.42 (m, 15H), 1.60 (m, 10H), 1.97 (m, 8H), 3.14 (s, 3H), 3.23 (s, 3H), 4.29 (m, 8H), 7.67 (s, 1H), 7.68 (s, 1H), 7.84 (dd, 1H, *J* = 8.6, 1.9 Hz), 8.31 (m, 2H), 8.70 (s, 1H), 8.74 (s, 1H) ppm; ^13^C NMR (151 MHz, CDCl_3_): δ 166.29, 152.32, 152.06, 149.56, 149.46, 143.38, 143.22, 142.77, 140.58, 132.03, 130.41, 129.27, 128.24, 127.19, 126.68, 123.57, 123.46, 108.99, 108.79, 106.45, 106.32, 69.66, 69.59, 69.18, 69.16, 61.45, 31.67, 29.33, 29.31, 29.25, 25.85, 25.82, 22.67, 22.66, 14.45, 14.07, 14.05; UV-vis (λ_max_(log ε), CHCl_3_): 286(4.70), 336(4.53), 414(4.16); FT-IR (neat): 1608 cm^−1^ (ν: C=O); HRMS (ESI+): *m*/*z* calcd for C_48_H_69_N_3_O_5_^+^: 752.4997, found: 752.4997.

Compound **6a** was prepared according to Method 1 and purified by recrystallization from acetonitrile to afford red crystals in 85% yield. ^1^H NMR (400 MHz, CDCl_3_): δ 0.95 (m, 13H), 1.42 (m, 18H), 1.61 (m, 7H), 1.99 (m, 8H), 4.08 (m, 4H), 4.31 (m, 8H), 4.61 (m, 4H), 7.71 (s, 2H), 8.36 (m, 2H), 8.76 (s, 2H), 9.05 (d, 1H, *J* = 1.3 Hz) ppm; ^13^C NMR (151 MHz, CDCl_3_): δ 170.80, 152.08, 152.01, 149.49, 142.65, 142.60, 141.63, 140.66, 136.35, 129.68, 127.78, 127.73, 126.85, 126.72, 123.62, 123.58, 108.88, 108.79, 106.45, 106.43, 69.64, 69.19, 69.15, 39.81, 35.54, 31.67, 29.71, 29.32, 29.25, 25.85, 25.82, 22.66, 14.07, 14.05; FT-IR (neat): 1726 cm^−1^ (ν: C=O); HRMS (ESI+): *m*/*z* calcd for C_47_H_65_N_2_O_7_^+^: 769.4786, found: 769.4786.

Compound **6c** was prepared according to Method 1 and purified by recrystallization from acetonitrile to afford red crystals in 78% yield. ^1^H NMR (400 MHz, CDCl_3_): δ 0.95 (m, 13H), 1.42 (m, 17H), 1.61 (m, 12H), 1.96 (m, 2H), 2.17 (9H), 3.70 (t, 2H, *J* = 6.5 Hz), 4.33 (m, 8H), 4.47 (t, 2H, *J* = 6.6 Hz), 7.73 (s, 2H), 8.37 (m, 2H), 8.80 (s, 1H), 8.81 (2, 1H), 9.04 (d, 1H, *J* = 1.6 Hz) ppm; ^13^C NMR (151 MHz, CDCl_3_): δ 170.80, 152.08, 152.01, 149.49, 142.65, 142.60, 141.63, 140.66, 136.35, 129.68, 127.78, 127.73, 126.85, 126.72, 123.62, 123.58, 108.88, 108.79, 106.45, 106.43, 69.64, 69.19, 69.15, 39.81, 35.54, 31.67, 29.71, 29.32, 29.25, 25.85, 25.82, 22.66, 14.07, 14.05; FT-IR (neat): 1722 cm^−1^ (ν: C=O); HRMS (ESI+): *m*/*z* calcd for C_51_H_73_N_2_O_7_^+^: 825.5413, found: 825.5412.

Compound **3** was prepared according to Method 2 and purified by column chromatography using dichloromethane as eluent (*R*_f_ = 0.43) to afford orange crystals in 27% yield and then further purified via recrystallization from hexanes. ^1^H NMR (400 MHz, CDCl_3_): δ 0.95 (m, 11H), 1.42 (m, 15H), 1.47 (s, 3H), 1.49 (s, 3H), 1.59 (m, 10H), 1.98 (m, 8H), 4.29 (t, 4H, *J* = 6.5 Hz), 4.36 (dd, 4H, *J* = 12.3, 6.4 Hz), 5.39 (m, 1H), 7.74 (s, 2H), 8.37 (m, 2H), 8.81 (d, 2H, *J* = 4.1 Hz), 9.04 (d, 1H, *J* = 1.6 Hz) ppm; ^13^C NMR (151 MHz, CDCl_3_): δ 166.29, 152.32, 152.06, 149.56, 149.46, 143.38, 143.22, 142.77, 140.58, 132.03, 130.41, 129.27, 128.24, 127.19, 126.68, 123.57, 123.46, 108.99, 108.79, 106.45, 106.32, 69.66, 69.59, 69.18, 69.16, 61.45, 31.67, 29.33, 29.31, 29.25, 25.85, 25.82, 22.67, 22.66, 14.45, 14.07, 14.05; FT-IR (neat): 1723 cm^−1^ (ν: C=O); HRMS (ESI+): *m*/*z* calcd for C_48_H_67_N_2_O_6_^+^: 767.4994, found: 767.4994.

Compound **4** was prepared according to Method 2 and purified by recrystallization from acetonitrile to afford red crystals in 70% yield. ^1^H NMR (400 MHz, CDCl_3_): δ 0.95 (m, 13H), 1.43 (m, 15H), 1.61 (m, 10H), 1.98 (m, 8H), 4.32 (m 8H), 7.34 (m, 3H), 7.49 (m, 2H), 7.72 (s, 2H), 8.47 (m, 2H), 8.80 (s, 2H), 9.23 (d, 1H, *J* = 1.8 Hz) ppm; ^13^C NMR (151 MHz, CDCl_3_): δ 166.29, 152.32, 152.06, 149.56, 149.46, 143.38, 143.22, 142.77, 140.58, 132.03, 130.41, 129.27, 128.24, 127.19, 126.68, 123.57, 123.46, 108.99, 108.79, 106.45, 106.32, 69.66, 69.59, 69.18, 69.16, 61.45, 31.67, 29.33, 29.31, 29.25, 25.85, 25.82, 22.67, 22.66, 14.45, 14.07, 14.05; UV-vis (λ_max_(log ε), CHCl_3_): 290(4.75), 342(4.66), 422(4.33); FT-IR (neat): 1738 cm^−1^ (ν: C=O); HRMS (ESI+): *m*/*z* calcd for C_51_H_65_N_2_O_6_^+^: 801.4838, found: 801.4837.

Compound **5** was prepared according to Method 2 and purified by recrystallization from acetonitrile to afford red crystals in 33% yield. ^1^H NMR (400 MHz, CDCl_3_): δ 0.95 (m, 11H), 1.43 (m, 16H), 1.60 (m, 14H), 1.98 (m, 13H), 4.32 (m 8H), 5.32 (m, 1H), 7.71 (s, 2H), 8.37 (m, 2H), 8.79 (s, 2H), 9.02 (d, 1H, *J* = 1.5 Hz) ppm; ^13^C NMR (151 MHz, CDCl_3_): δ 166.29, 152.32, 152.06, 149.56, 149.46, 143.38, 143.22, 142.77, 140.58, 132.03, 130.41, 129.27, 128.24, 127.19, 126.68, 123.57, 123.46, 108.99, 108.79, 106.45, 106.32, 69.66, 69.59, 69.18, 69.16, 61.45, 31.67, 29.33, 29.31, 29.25, 25.85, 25.82, 22.67, 22.66, 14.45, 14.07, 14.05; UV-vis (λ_max_(log ε), CHCl_3_): 288(4.68), 339(4.57), 419(4.24); FT-IR (neat): 1722 cm^−1^ (ν: C=O); HRMS (ESI+): *m*/*z* calcd for C_51_H_71_N_2_O_6_^+^: 807.5307, found: 807.5307.

## 4. Conclusions

In the current work, we have demonstrated that dibenzophenazine esters are a versatile class of compounds that form columnar hexagonal phases over extremely broad temperature ranges. These systems tolerate considerable variation in structure without the loss of liquid crystallinity and without significantly altering the electronic structure of the core. Such features may make these compounds promising candidates as organic semiconductors, where fine-tuning self-assembly independently of the electronic properties is an important criterion. We have further observed that replacing the ester functionality with an amide group substantially lowers the clearing temperature, underscoring the importance of electronic considerations in the design of new mesogens. A common feature of all molecules examined in this study was their tendency to supercool. While most compounds crystallized quickly, the phenyl ester **4** retained liquid crystalline ordering for prolonged periods at room temperature, pointing the way to the design of stable glass-forming mesogens. Such studies are currently being undertaken in our laboratory.

## References

[1] Kaafarani B.R. (2011). Discotic liquid crystals for opto-electronic applications. Chem. Mater..

[2] Laschat S., Baro A., Steinke N., Giesselmann F., Hägele C., Scalia G., Judele R., Kapatsina E., Sauer S., Schreivogel A. (2007). Discotic liquid crystals: From tailor-made synthesis to plastic electronics. Angew. Chem. Int. Ed..

[3] Imrie C.T., Henderson P.A. (2007). Liquid crystal dimers and higher oligomers: Between monomers and polymers. Chem. Soc. Rev..

[4] Bushby R.J., Kawata K. (2011). Liquid crystals that affected the world: Discotic liquid crystals. Liq. Cryst..

[5] Kumar S. (2006). Self-organization of disc-like molecules: Chemical aspects. Chem. Soc. Rev..

[6] Kumar S. (2005). Triphenylene-based discotic liquid crystal dimers, oligomers and polymers. Liq. Cryst..

[7] Keuker-Baumann S., Bock H., Sala F.D., Benning S.A., Haßheider T., Frauenheim T., Kitzerow H.-S. (2001). Absorption and luminescence spectra of electroluminescent liquid crystals with triphenylene, pyrene and perylene units. Liq. Cryst..

[8] Hassheider T., Benning S.A., Kitzerow H.-S., Achard M.-F., Bock H. (2001). Color-tuned electroluminescence from columnar liquid crystalline alkyl arenecarboxylates. Angew. Chem. Int. Ed..

[9] Benning S., Kitzerow H.-S., Bock H., Achard M.-F. (2000). Fluorescent columnar liquid crystalline 3,4,9,10-tetra-(n-alkoxycarbonyl)-perylenes. Liq. Cryst..

[10] Ito S., Wehmeier M., Brand J.D., Kübel C., Epsch R., Rabe J.P., Müllen K. (2000). Synthesis and self-assembly of functionalized hexa-peri-hexabenzocoronenes. Chem. Eur. J..

[11] Foster E.J., Lavigueur C., Ke Y.-C., Williams V.E. (2005). Self-assembly of hydrogen-bonded molecules: Discotic and elliptical mesogens. J. Mater. Chem..

[12] Bozek K.J.A., Williams V.E. (2014). Folded discotic dimers. Soft Matter.

[13] Hansch C., Leo A., Unger S.H., Kim K.H., Nikaitani D., Lien E.J. (1973). Aromatic substituent constants for structure-activity correlations. J. Med. Chem..

[14] Szydłowska J., Krzyczkowska P., Salamończyk M., Górecka E., Pociecha D., Maranowski B., Krówczyński A. (2013). Gelling and fluorescent mesogens of quinoxaline analogs. J. Mater. Chem. C.

[15] Ong C.W., Hwang J.-Y., Tzeng M.-C., Liao S.-C., Hsu H.-F., Chang T.-H. (2007). Dibenzo[*a*,*c*]phenazine with six-long alkoxy chains to probe optimization of mesogenic behavior. J. Mater. Chem..

[16] Ong C.W., Hwang C.-Y., Liao S.-C., Pan C.-H., Chang T.-H. (2009). Indent and bulge-discogens for controlling the columnar mesophase. J. Mater. Chem..

[17] Kumar S. (2010). Chemistry of Discotic Liquid Crystals: From Monomers to Polymers.

[18] Lavigueur C., Foster E.J., Williams V.E. (2008). Self-assembly of discotic mesogens in solution and in liquid crystalline phases: Effects of substituent position and hydrogen bonding. J. Am. Chem. Soc..

[19] Collard D.M., Lillya C.P. (1989). Control of thermal phase behavior of disklike molecules by modification of side-chain structure. J. Am. Chem. Soc..

[20] Collard D.M., Lillya C.P. (1991). Structure–property relationships for the thermal phase behavior of discotic liquid crystals: The effect of branching and unsaturation in the side chains of disklike molecules. J. Am. Chem. Soc..

[21] Varshney S.K., Prasad V., Takezoe H. (2011). Room-temperature discotic cholesteric and nematic phases: influence of 3,7-dimethyloctane peripheral chain on the molecular self-assembly of radial polyalkynylbenzene. Liq. Cryst..

[22] Stackhouse P.J., Hird M. (2008). Influence of branched chains on the mesomorphic properties of symmetrical and unsymmetrical triphenylene discotic liquid crystals. Liq. Cryst..

[23] Paleos C.M., Tsiourvas D. (2001). Supramolecular hydrogen-bonded liquid crystals. Liq. Cryst..

[24] Wang B.Q., Zhao K.Q., Hu P., Yu W.H., Gao C.Y., Shimizu Y. (2007). Tuning hydrogen-bonding with amide groups for stable columnar mesophases of triphenylene discogens. Mol. Cryst. Liq. Cryst..

[25] Boden N., Bushby R.J., Lu Z.B. (1998). A rational synthesis of polyacrylates with discogenic side groups. Liq. Cryst..

[26] Grafe A., Janietz D., Frese T., Wendorff J.H. (2005). Star-shaped discotic oligomesogens based on radial pentakisphenylethynylbenzene moieties. Chem. Mater..

[27] Foster E.J., Jones R.B., Lavigueur C., Williams V.E. (2006). Structural factors controlling the self-assembly of columnar liquid crystals. J. Am. Chem. Soc..

[28] Lavigueur C., Foster E.J., Williams V.E. (2008). A simple and inexpensive capillary furnace for variable-temperature X-ray diffraction. J. Appl. Crystallogr..

